# Genome metabolome integrated network analysis to uncover connections between genetic variants and complex traits: an application to obesity

**DOI:** 10.1098/rsif.2013.0908

**Published:** 2014-05-06

**Authors:** Beatriz Valcárcel, Timothy M. D. Ebbels, Antti J. Kangas, Pasi Soininen, Paul Elliot, Mika Ala-Korpela, Marjo-Riitta Järvelin, Maria de Iorio

**Affiliations:** 1 Department of Epidemiology and Biostatistics, School of Public Health, MRC-HPA Centre for Environment and Health, Faculty of Medicine, Imperial College London, London, UK; 2 Computational and Systems Medicine, Department of Surgery and Cancer, Imperial College London, London, UK; 3 Computational Medicine, University of Oulu, Oulu, Finland; 4 Institute of Health Sciences, University of Oulu, Oulu, Finland; 5 NMR Metabolomics Laboratory, School of Pharmacy, University of Eastern Finland, Kuopio, Finland; 6 Computational Medicine, School of Social and Community Medicine, University of Bristol, Bristol, UK; 7 Biocenter Oulu, University of Oulu, Oulu, Finland; 8 Department of Lifecourse and Services, National Institute of Health and Welfare, Oulu, Finland; 9 Department of Statistical Science, University College London, London, UK

**Keywords:** correlation analysis, differential networks, genome-wide association analysis, metabolomics, GEMINi

## Abstract

Current studies of phenotype diversity by genome-wide association studies (GWAS) are mainly focused on identifying genetic variants that influence level changes of individual traits without considering additional alterations at the system-level. However, in addition to level alterations of single phenotypes, differences in association between phenotype levels are observed across different physiological states. Such differences in molecular correlations between states can potentially reveal information about the system state beyond that reported by changes in mean levels alone. In this study, we describe a novel methodological approach, which we refer to as genome metabolome integrated network analysis (GEMINi) consisting of a combination of correlation network analysis and genome-wide correlation study. The proposed methodology exploits differences in molecular associations to uncover genetic variants involved in phenotype variation. We test the performance of the GEMINi approach in a simulation study and illustrate its use in the context of obesity and detailed quantitative metabolomics data on systemic metabolism. Application of GEMINi revealed a set of metabolic associations which differ between normal and obese individuals. While no significant associations were found between genetic variants and body mass index using a standard GWAS approach, further investigation of the identified differences in metabolic association revealed a number of loci, several of which have been previously implicated with obesity-related processes. This study highlights the advantage of using molecular associations as an alternative phenotype when studying the genetic basis of complex traits and diseases.

## 1. Introduction

With the development of high-throughput genotyping technology, genome-wide association analysis (GWAS) has become a powerful tool to examine the genetic basis of many complex traits and common diseases. These studies have substantially increased our knowledge of genes that influence phenotypic variations. For instance, in obesity genetics, substantial progress has been made with the discovery of at least 50 common and rare variants influencing obesity-related traits [1–6]. Yet, despite this great success, a large proportion of the genetic variation contributing to the observed variability in phenotypes still remains unmapped [7,8]. In trying to discover the ‘missing variants’, a number of strategies have been proposed [9]. It has been suggested that considering alternative models of genetic effects and their influence on complex traits can help to discover some of the missing variants [9–12].

In an earlier work [13], we highlighted the importance of using molecular association patterns and their differences in relation to different physiological conditions to investigate the biological features of phenotype variation. We developed a network-based method for the differential analysis of molecular associations and illustrated its use in the context of prediabetes. Based on this analysis, we were able to identify key differences in lipoprotein metabolism known to be related to the early development of diabetic dyslipidaemias. Importantly, these changes were not identified by comparing changes in mean concentration levels at the early phase of disease development. These results indicate that differences in molecular associations might provide access to unique aspects of the underlying genetic and molecular mechanisms, being therefore a complementary tool to unravel the biological basis of phenotype variation.

In this study, we hypothesize that some of the undetected variants might be associated not with changes in mean levels of a phenotype, as studied by conventional GWAS, but with alterations that occur at a higher system-level. To investigate the potential use of differences in molecular dependencies to uncover genetic variants associated with phenotype variation, we propose a novel analytical approach to integrate metabolic and genetic information, which we refer to as genome metabolome integrated network analysis (GEMINi). A simulation study is initially presented to assess the performance of the proposed methodology. We then demonstrate the use of our approach by investigating metabolic changes in relation to obesity using two independent population-based cohorts, totalling to over 5000 individuals. A recently established serum NMR metabolomic platform [14] was applied to quantify 38 systemic metabolites that represent various key metabolic pathways [15].

The proposed GEMINi approach identifies potentially new genetic components of obesity. Differences in association between serum metabolites were identified, comprising several metabolites linked by previous studies to the development of obesity-related disorders. Metabolites involved in such differential connections included all sizes of very-low-density lipoprotein particles (VLDL) as well as several low-molecular-weight metabolites. Further investigation of these phenotypic differences revealed a set of genetic variants significantly associated with these differential metabolic dependencies. Several of the associated genes have been previously implicated in obesity-related traits. Besides the GEMINi approach, we also performed two standard analyses. First, we looked for associations between genetic variants and body mass index (BMI) by a conventional GWAS approach. Second, we looked for associations between mean levels of the metabolites and BMI. Using the conventional GWAS approach, we found no associations at a genome-wide significance level between single nucleotide polymorphisms (SNPs) and BMI. These results highlight the advantage of using differences in statistical association between different molecules as an additional phenotype in the study of common trait variation and demonstrate the applicability of our approach to uncover further molecular factors potentially involved in the pathogenesis of complex traits.

## 2. Material and methods

### 2.1. Study populations

We used data from two large population-based cohorts, the Northern Finland Birth Cohort 1966 (NFBC1966) and the Northern Finland Birth Cohort 1986 (NFBC1986). The methods and aims of these studies have been published previously [16]. In brief, the NFBC1966 includes 12 068 children with expected dates of birth falling in between 1 January and 31 December in the two northernmost provinces of Finland, Oulu and Lapland. Data were collected since pregnancy and supplemented at the age of 1, 14 and 31 years. Blood samples were taken when individuals were 31 years old. The NFBC1986 includes 9432 live born children with expected dates of birth between 1 July 1985 and 30 June 1986 also in the above area. The cohort has been monitored since early pregnancy until adolescence. All those alive with known address were invited to a clinical examination at the age of 15–16 years when blood samples were taken. The blood collections were drawn after overnight fasting and the samples stored at −80°C. The University of Oulu Ethics Committee and the Ethical Committee of Northern Ostrobothnia Hospital District have approved the studies, and all participants provided written informed consent.

### 2.2. Phenotype measures

For the differential network analysis, individuals were classified into two groups: obese and non-obese according to their BMI values. For the NFBC1966, individuals were classified as obese if their BMI exceeded 30 kg m^−2^. Individuals were classified as non-obese if their BMI was equal or lower than 25 kg m^−2^ and equal or greater than 18.5 kg m^−2^. For the NFBC1986, individuals were classified as obese and non-obese by calculating the age- and sex-specific BMI percentiles. The non-obese group includes females with measures of BMI equal or lower than 24.05 kg m^−2^ and equal or greater than 17.4 kg m^−2^ and males with BMI equal or lower than 24.2 kg m^−2^ and equal greater than 17 kg m^−2^ (fifth percentile to less than the 85th percentile). The obese group includes female with BMI greater than 27.5 kg m^−2^ and males with BMI greater than 28.2 kg m^−2^ (greater than the 95th percentile). Individuals were excluded from analysis based on the following criteria: non-fasting, diagnosed type I or II diabetes, pregnancy, lipid-lowering medication, missing data on glucose and/or weight measures and/or height. The sample size for NFBC1966 is 3464 subjects (*N*_non-obese_ = 3023 and *N*_obese_ = 441) and for NFBC1986 it is 3791 subjects (*N*_non-obese_ = 3565 and *N*_obese_ = 226). The clinical characteristics of the study groups are presented in the electronic supplementary material, tables S1 and S2.

### 2.3. Metabolic data

The metabolic data for the two cohorts were acquired using the same high-throughput serum NMR metabolomics platform the details of which have been described previously [8,14]. Metabolites were selected according to feasibility of quantification by NMR and to allow a consistent analysis across the two cohorts. Table 1 shows the 38 metabolic measures included in the GEMINi analysis, representing a wide variety of metabolic processes, such as amino acid, energy and lipoprotein metabolism.

**Table 1.** Linear regression analysis between body mass index (BMI) and the serum metabolite measures. *p*, *p*-value. A *p*-value given in italics indicates a statistically significant association between BMI and a metabolite measure with a Bonferroni-corrected threshold *p* < 0.001/*M*, where *M* = 38 is the total number of metabolites included in the analyses; *β*, standardized beta coefficient; *Q*, *q*-value (Benjamini & Hochberg [17]).

metabolic measures	abbreviation	NFBC1966			NFBC1986		
		*β*	*p*	*Q*	*β*	*p*	*Q*
total lipids in chylomicrons and extremely large VLDL	XXL-VLDL-L	0.21	*3.99 × 10^−50^*	9.48 × 10^−50^	0.28	*1.58 × 10^−79^*	6.67 × 10^−79^
total lipids in very large VLDL	XL-VLDL-L	0.21	*6.39 × 10^−50^*	1.43 × 10^−49^	0.29	*4.58 × 10^−88^*	3.48 × 10^−87^
total lipids in large VLDL	L-VLDL-L	0.22	*4.01 × 10^−55^*	1.52 × 10^−54^	0.30	*2.58 × 10^−93^*	2.45 × 10^−92^
total lipids in medium VLDL	M-VLDL-L	0.24	*1.02 × 10^−62^*	5.54 × 10^−62^	0.31	*1.72 × 10^−100^*	3.27 × 10^−99^
total lipids in small VLDL	S-VLDL-L	0.26	*3.08 × 10^−73^*	3.90 × 10^−72^	0.32	*1.89 × 10^−102^*	7.18 × 10^−101^
total lipids in very small VLDL	XS-VLDL-L	0.21	*3.38 × 10^−50^*	8.56 × 10^−50^	0.23	*2.01 × 10^−53^*	5.09 × 10^−53^
total lipids in IDL	IDL-L	0.18	*1.28 × 10^−37^*	2.32 × 10^−37^	0.20	*9.22 × 10^−38^*	1.75 × 10^−37^
total lipids in large LDL	L-LDL-L	0.19	*3.74 × 10^−41^*	7.11 × 10^−41^	0.21	*4.28 × 10^−44^*	8.56 × 10^−44^
total lipids in medium LDL	M-LDL-L	0.21	*2.24 × 10^−49^*	4.73 × 10^−49^	0.23	*1.36 × 10^−52^*	3.23 × 10^−52^
total lipids in small LDL	S-LDL-L	0.24	*4.80 × 10^−64^*	3.65 × 10^−63^	0.25	*1.99 × 10^−63^*	6.30 × 10^−63^
total lipids in very large HDL	XL-HDL-L	−0.11	*5.37 × 10^−14^*	8.87 × 10^−14^	−0.25	*3.73 × 10^−57^*	1.01 × 10^−56^
total lipids in large HDL	L-HDL-L	−0.23	*5.80 × 10^−53^*	1.70 × 10^−52^	−0.27	*5.21 × 10^−69^*	1.98 × 10^−68^
total lipids in medium HDL	M-HDL-L	−0.07	*3.03 × 10^−7^*	4.11 × 10^−7^	0.03	4.58 × 10^−2^	0.047
total lipids in small HDL	S-HDL-L	0.06	*3.26 × 10^−5^*	4.27 × 10^−5^	0.17	*5.13 × 10^−30^*	8.86 × 10^−30^
apolipoprotein A–I	ApoA1	−0.05	6.83 × 10^−4^	8.11 × 10^−4^	−0.06	3.16 × 10^−4^	3.64 × 10^−4^
apolipoprotein B	ApoB	0.25	*1.04 × 10^−63^*	6.59 × 10^−63^	0.30	*2.15 × 10^−87^*	1.36 × 10^−86^
mean diameter for VLDL particles	VLDL-D	0.21	*4.13 × 10^−47^*	8.26 × 10^−47^	0.21	*1.22 × 10^−45^*	2.58 × 10^−45^
mean diameter for LDL particles	LDL-D	−0.11	*7.92 × 10^−14^*	1.25 × 10^−13^	−0.09	*5.74 × 10^−9^*	8.08 × 10^−9^
mean diameter for HDL particles	HDL-D	−0.25	*1.04 × 10^−58^*	4.39 × 10^−58^	−0.32	*1.41 × 10^−94^*	1.79 × 10^−93^
3-hydroxybutyrate	bOHBut	−0.02	0.102	0.111	−0.07	*1.05 × 10^−6^*	1.38 × 10^−6^
acetate	Ace	−0.03	*3.11 × 10^−2^*	0.035	−0.07	*1.29 × 10^−5^*	1.63 × 10^−5^
acetoacetate	AcAce	−0.01	0.615	0.615	−0.05	1.31 × 10^−3^	1.42 × 10^−3^
alanine	Ala	0.13	*9.29 × 10^−21^*	1.60 × 10^−20^	0.10	*2.68 × 10^−12^*	4.07 × 10^−12^
citrate	Cit	−0.04	2.84 × 10^−3^	3.27 × 10^−3^	−0.15	*1.15 × 10^−23^*	1.90 × 10^−23^
creatinine	Crea	−0.02	0.164	0.173	0.07	*2.62 × 10^−5^*	3.21 × 10^−5^
glucose	Glc	0.09	*7.12 × 10^−10^*	1.00 × 10^−9^	0.03	2.44 × 10^−2^	2.58 × 10^−2^
glutamine	Gln	−0.06	*8.67 × 10^−5^*	1.10 × 10^−4^	−0.09	*8.36 × 10^−8^*	1.13 × 10^−7^
glycerol	Glol	0.21	*4.70 × 10^−52^*	1.28 × 10^−51^	0.11	*8.24 × 10^−13^*	1.30 × 10^−12^
glycoprotein acetyls, mainly a1-acid glycoprotein	Gp	0.22	*2.19 × 10^−60^*	1.04 × 10^−59^	0.28	*9.63 × 10^−82^*	4.57 × 10^−81^
histidine	His	0.05	5.99 × 10^−4^	7.34 × 10^−4^	0.10	*1.02 × 10^−11^*	1.49 × 10^−11^
isoleucine	Ile	0.29	*2.61 × 10^−82^*	4.96 × 10^−81^	0.26	*9.12 × 10^−65^*	3.15 × 10^−64^
lactate	Lac	0.10	*4.30 × 10^−12^*	6.54 × 10^−12^	−0.05	8.56 × 10^−4^	9.57 × 10^−4^
leucine	Leu	0.26	*2.76 × 10^−67^*	2.62 × 10^−66^	0.25	*1.74 × 10^−60^*	5.09 × 10^−60^
phenylalanine	Phe	0.28	*5.27 × 10^−93^*	2.00 × 10^−91^	0.29	*2.43 × 10^−85^*	1.32 × 10^−84^
pyruvate	Pyr	0.09	*5.57 × 10^−11^*	8.14 × 10^−11^	0.06	*5.38 × 10^−5^*	6.39 × 10^−5^
tyrosine	Tyr	0.22	*6.87 × 10^−54^*	2.18 × 10^−53^	0.23	*5.24 × 10^−51^*	1.17 × 10^−50^
urea	Urea	0.01	0.503	0.517	−0.02	1.76 × 10^−1^	0.176
valine	Val	0.23	*6.38 × 10^−54^*	2.18 × 10^−53^	0.19	*5.84 × 10^−32^*	1.06 × 10^−31^

### 2.4. Genetic data

Only the NFBC1966 were used for the genetic analysis, because genome-wide data for the NFBC1986 were not available. Details for the genotyping procedure and quality control are presented in reference [16]. In brief, all DNA samples were prepared and genotyped by the Broad Institute Biological Sample Repository. Genotyping was performed using the Illumina Infinium 370cnvDuo array. The per-sample quality control process included analysis of duplicate samples, sex discrepancy, sample contamination and relatedness. Of the related pairs that shared over 20% of the genome identical by descent, the individuals with less complete genotype data were excluded. Markers were excluded if the call rate in was less than 95%, if the minor allele frequency was less than 1% and if the *p*-value from a Hardy–Weinberg equilibrium test was *p* < 1 × 10^−4^. The final genetic dataset included 4815 individuals and 318 443 SNPs

### 2.5. Statistical methods

In this study, we perform and compare two statistical procedures. First, we perform a standard analysis of the associations between metabolic levels and BMI using univariate linear regression and between genetic variants and BMI following a conventional GWAS approach. The second approach, which we refer to as GEMINi, is a combination of (i) differential network analysis of metabolic associations, and (ii) genome-wide correlation (GWC) study. We assess the performance of the proposed GEMINi methodology by performing a simulation study (see the electronic supplementary material). All analyses were adjusted for sex differences in serum metabolite levels.

#### 2.5.1. Conventional analysis of association between obesity and metabolic–genetic data

A conventional GWAS approach is used to test for associations between genetic variants and BMI. The analysis is performed following the standard single-SNP approach where SNPs are tested one at a time. Associations were investigated using linear regression assuming an additive effect on the trait and including sex as a covariate. Significant associations between genetic variants and BMI measures were assessed by setting genome-wide significance to *p* < 5 × 10^−8^ [18].

We use linear regression to test the effect of serum metabolites on BMI. The analyses are performed following a univariate approach where metabolites are tested one at a time. We included sex as a covariate to correct for sex differences in serum metabolic profiles. To identify significant associations between serum metabolites and BMI, we use a conservative Bonferroni-corrected significance level, *p* < 0.01/*M*, where *M* = 38 denotes the total number of serum metabolite measures.

The above analyses were conducted including all the individuals for which genetic, metabolic and BMI data were available (*N* = 4346).

#### 2.5.2. Genome metabolome integrated network analysis

**Figure 1.**
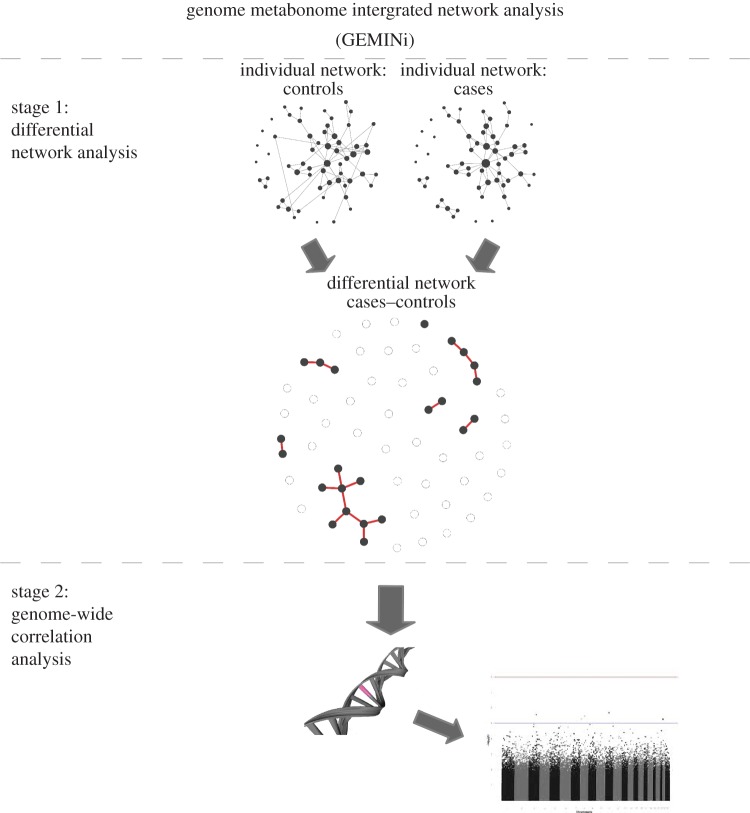
An outline of the GEMINi methodology. (Online version in colour.)

An outline of the GEMINi methodology is presented in figure 1. The method consists of two stages: (i) construction of the differential network, and (ii) a genome-wide correlation analysis (GWCA). We start by performing a differential network analysis that allows us to test whether the pattern of pairwise associations between metabolites is the same in two physiological groups (e.g. non-obese and obese) or whether it significantly differs across groups. To eliminate the confounding effect of sex on the serum metabolites, the data used for this analysis are the residuals from a linear regression model of each metabolite on sex. To build the differential networks, we use the same methodology presented in reference [13]. Briefly, the underlying interdependencies between metabolites are initially measured for each of the two physiological groups using shrinkage estimates of partial correlations [19]. To test whether the association between metabolites significantly differs between groups, we perform a two-sample permutation test. We used 100 000 permutations in our analysis. If the partial correlations between two given metabolites are significantly different between the two physiological groups, then we draw an edge in the differential network. The connections included in the differential network are defined by setting a cut-off on the two-tailed *p*-value. The power to estimate correlations is lower than the one to estimate a change in mean levels, therefore, to infer the differential network we set an uncorrected threshold, *p* < 0.01. To validate the differential network analysis results, we compare the network structure between the two cohorts. The replicated results between cohorts are further investigated in the next step of the analysis.

In the second step, we perform a GWCA to identify genetic variants associated with differences in metabolic associations. As for the standard GWAS study, all individuals for whom genetic data are available were included in the analysis. To find the desired associations, we first classify individuals according to the number of copies of the less frequent allele carried, giving genotype groups A (0 copies) and B (one or two copies). SNPs are tested one at a time. Subsequently, the correlation between metabolites *m*_1_ and *m*_2_ is calculated for each group, *r*_A_ and *r*_B,_ and differences in correlation between groups A and B is tested. As in the differential network analysis, we eliminate the confounding effect of sex on the serum metabolites by using the residuals of a linear regression of the metabolite level on sex. To test whether the two correlation coefficients, *r*_A_ and *r*_B_ are the same, we use the *z* transform method as described in reference [20]. To correct for multiple testing, significant associations between genetic variants and variations in metabolic associations are determined using the genome-wide significance threshold *p* < 5 × 10^−8^/*D*, which corresponds to a genome-wide significance level adjusted with the number of differential connections (*D*) identified in step one. To assess the biological significance of our findings, identified SNPs are assigned to the nearest gene (maximum distance 1 Mb).

All analyses were conducted in R v. 2.14.1, apart from the conventional GWAS that was carried out in SNPtest v. 2.0. Computation of the partial correlation matrix was performed using the R package GeneNet. The differential networks were built and visualized using the R package igraph. The package psych in R was used to test for differences between correlation coefficients.

## 3. Results and discussion

### 3.1. Conventional analysis of metabolic and genetic data

#### 3.1.1. Genome-wide association study analysis of BMI

We performed a standard GWAS analysis where associations between genetic variants and BMI (as a continuous variable) were tested. A Manhattan plot of the *p*-values obtained from the GWAS analysis is presented in the electronic supplementary material, figure S1. Although no SNPs showed association at the genome-wide significance threshold (*p* < 5 × 10^−8^), several notable associations were found, of which the most significant was a variant within a gene encoding for a transcriptional factor-activating enhancer-binding protein two-beta (TFAP2B) (rs987237, 6p12.3, *p* < 1.53 × 10^−6^; see the electronic supplementary material, table S3) expressed in adipose tissue. The TFAP2B-rs987237 genetic variant has been previously associated with a number of metabolic disorders, including development of T2DM [21], BMI [22] and other BMI-related phenotypes such as waist circumference [4]. In addition, several SNPs localized within regions previously associated with BMI showed associations at a significance threshold of *p* < 0.001. The results for a list of loci localized within known obesity genes are presented in the electronic supplementary material, table S3.

Owing to the stringent genome-wide significance threshold (*p* < 5 × 10^−8^), application of the GWAS approach to a single population frequently fails to identify statistically significant associations. Despite the low *p*-values for some of the previously reported obesity-related genes, we did not reach the genome-wide significance threshold (*p* < 5 × 10^−8^) which might indicate the lack of power in this cohort to identify loci with key roles in determining variations in BMI.

#### 3.1.2. Association analysis between serum metabolites and body mass index

We performed an inspection of the data using linear regression to explore associations between serum metabolites and BMI. Results for the two cohorts are presented in table 1. To facilitate comparison of the cohorts, scatter plots for the estimated values are presented in the electronic supplementary material, figure S2. The analysis shows that all VLDL subclasses, intermediate density lipoprotein (IDL), all low-density lipoprotein subclasses (LDL)—apart from mean diameter for LDL particles—small high-density lipoprotein subclass (HDL) and apolipoprotein B are positively associated (*p* < 0.01/*M*) with BMI. Very large, large HDL and mean diameter for LDL and HDL particles are inversely associated with BMI in both cohorts. No association was found between BMI and apolipoprotein A in either of the cohorts. In NFBC1966, medium HDL is inversely associated with BMI, but shows no association in NFBC1986, which is potentially owing to the age difference between cohorts [23]. Tests of the association between BMI and low-molecular-weight metabolites show that alanine, glycerol, glycoprotein, isoleucine, leucine, phenyalanine, pyruvate, tyrosine and valine are positively associated with BMI in both cohorts, whereas glutamine is inversely associated with BMI. No association was found between BMI and acetoacetate and BMI and urea. We observe that glucose and lactate are positively associated with BMI, but these two metabolites show no association in NFBC1986. On the other hand, 3-hydroxybutyrate, acetate and citrate are negatively associated with BMI and creatinine and histidine are positively associated in NFBC1986, but shows no association in NFBC1966.

The large number of significant associations between BMI and the metabolite concentrations reflects the close relationship between obesity and systemic metabolism. However, it is not only the concentration levels of serum metabolites that can change owing to variations in total body mass, but also the dependencies between metabolites can be affected. To investigate how metabolic associations differ between non-obese and obese individuals and to what extent these differences can serve as an indicator of important molecular alteration between these physiological states, we use the GEMINi approach.

### 3.2. Genome metabolome integrated network analysis

#### 3.2.1. Simulation study

We carried out a simulation study to assess the performance of the GEMINi approach (see the electronic supplementary material). Four different scenarios were simulated by combining different values of (i) minor allele frequency, and (ii) differences in associations between variables across groups. These scenarios were created to cover the range of variation observed in the data. To illustrate the predictive power of GEMINi, the receiver operating characteristic (ROC) curve analysis was used for each of the two steps of the GEMINi approach (differential network and GWCA) and the area under the curves determined (AUC_DiffNet_ and AUC_GWCA_). Results are presented in the electronic supplementary material, figures S3 and S4.

We observe that for large differences in associations between variables across groups (simulation 1 and 2), the GEMINi approach shows perfect prediction value (AUC_DiffNet_ = 1.0 and AUC_GWCA_ = 1). With small differences in associations between variables across groups (simulation 3 and simulation 4), the accuracy of GEMINi drops, showing moderate prediction values. The area under the ROC curves for simulation 3, for the two steps of the GEMINi approach are AUC_DiffNet_ 0.7748 and AUC_GWCA_ 0.7793. For simulation 4, the area under the ROC curves are AUC_DiffNe_ = 0.6885 and AUC_GWCA_ = 0.7377.

Therefore, the GEMINi method shows a good performance in identifying differences in associations between a genetic variant and differences in pairwise associations between variables across different groups.

#### 3.2.2. Differential networks: non-obese and obese individuals

Differential network analysis is performed with the aim of determining the subset of metabolic associations that significantly differ between non-obese and obese individuals. We examine the same metabolic variables from the two independent cohorts (NFBC1966 and NFBC1986), and use estimates of partial correlation as measures of metabolic dependency. Metabolic associations that are consistently different across the two physiological conditions (obese and non-obese) in the two study cohorts are interpreted as obesity-related molecular characteristics and are further investigated in the GWCA.

**Figure 2.**
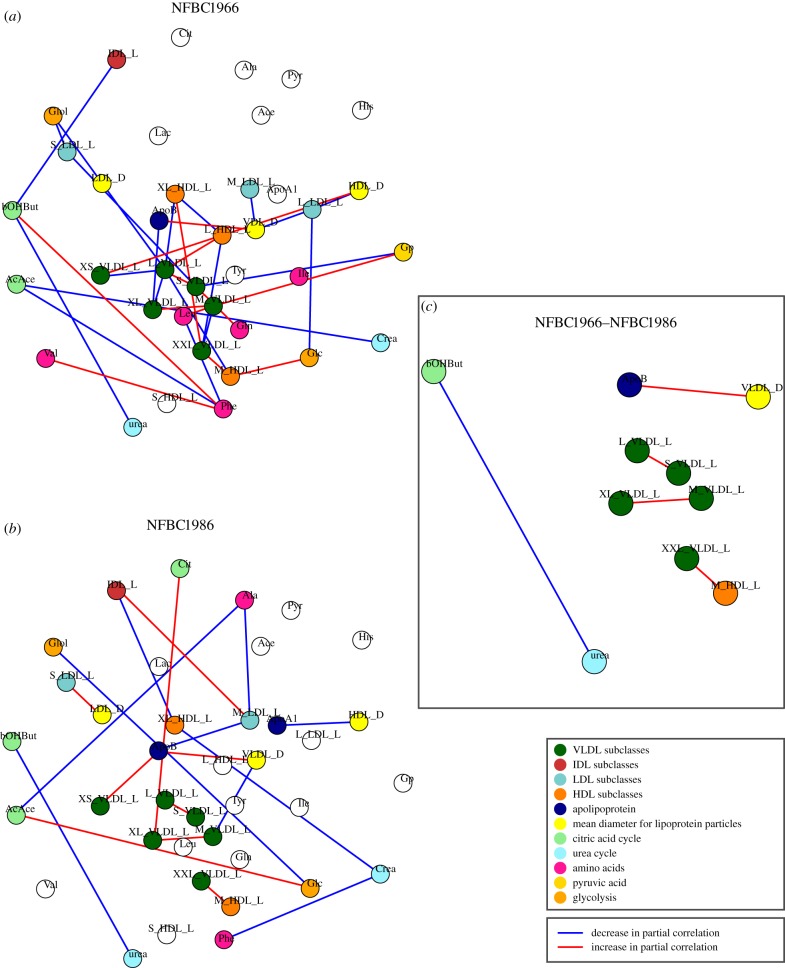
The differential analysis for BMI. (Online version in colour.)

The differential analysis for BMI is presented in figure 2. Each connection in the differential network indicates that a significant difference in the pairwise association between two metabolic variables is found between the two physiological conditions. To assess the robustness of differences in the serum metabolite associations, we compare the two differential networks (figure 2*c*). The differential network for NFBC1966 (figure 2*a*) consists of a set of 29 connected variables, which are organized in 36 pairwise differential interactions. The differential network for NFBC1986 (figure 2*b*) comprises a group of 25 differentially connected variables, which are organized in 19 pairwise differential interactions. We observe similar patterns of differences in the metabolic associations in these two differential networks. For both networks, the majority of the connected variables represent measures of VLDL (dark green nodes), LDL (light blue nodes), HDL (dark orange nodes) and amino acids (pink nodes). Five metabolites—pyruvate, histidine, lactate, tyrosine, total lipids in small HDL—show no connections in either of the two networks. Five overlapping connections are observed between the two networks (figure 2*c*). This overlap is significantly higher than expected at random with *p*-value = 1.97 × 10^−4^ from a hypergeometric test with *N* = 703 pairs, *M* = 36, *K* = 19, *x* = 5). The overlapping connections highlight differences in the pairwise associations between VLDL measures which occur throughout all size ranges, one measure of HDL (M-HDL-L) and two low-molecular-weight metabolites (urea and 3-hydroxybutyrate). Moreover, not only are a number of connected components overlapping between the two networks, but also the signs of the difference in partial correlations are identical (represented as connection colours). We observe that the correlation is significantly higher in the obese group for small VLDL and large VLDL, ApoB and mean diameter for VLDL, large VLDL and small VLDL, very large and medium VLDL and extremely large VLDL and medium HDL (red edges). On the other hand, we observe that partial correlation between the pair urea and 3-hydroxybutyrate is significantly lowered (blue edges) in the obese group.

A number of obesity-related differences in metabolic associations identified here are in line with prior studies on obesity-related traits. For instance, the central position and abundance of VLDL-related measures in the differential networks may reflect disregulation in VLDL metabolism. These alterations may result from the shift from small VLDL particles to enlarged VLDL [24]. There is also evidence to suggest that in obesity-related dyslipidaemia an overproduction of large VLDL particles is the initiator of all sequences of lipoprotein changes, resulting in a decrease in levels and particle size of HDL and smaller-denser LDL particles [25]. Previous studies on obesity-related dyslipidaemias have recognized a complex pattern of change in size and particle concentration within the major lipoprotein classes in patients with obesity, especially abdominal obesity [26,27]. These disorders are related to elevated VLDL triglycerides that trigger the shift from small VLDL to medium and large VLDL, increased levels of small LDL and reduction in concentration and size of HDL. These variations in lipoprotein particle size subclasses, which relate to dyslipidaemias associated with obesity, are captured by the differential network of lipoprotein associations.

The relatively large number of independently replicated results and the known relevance of the identified metabolites to obesity provide strong support for differential association analysis in improving our understanding of the biological mechanisms of disease. Assuming that these metabolic differences are related to obesity, we hypothesized that these measures could be used as an additional phenotype to identify genetic variants associated with obesity-related traits. To test this hypothesis, we performed a GWCA.

#### 3.2.3. Genome-wide correlation analysis of metabolic associations

The associations of 318 443 SNPs with the differences in metabolite–metabolite partial correlations were individually tested. Our analysis revealed 24 loci significantly associated with differences in associations between one pair of metabolites, namely total lipids in medium VLDL and very-large VLDL (*p* < 5 × 10^−8^/*D*; *D* = 5). The identified SNPs are localized in 18 genetic regions across 14 chromosomes. A brief description of the identified genetic variants, including their accession numbers, chromosomal positions, obtained *p*-values, along with candidate genes associated with the genetic marker is presented in table 2. The Manhattan plot of the *p*-values obtained from the GWCA is presented in the electronic supplementary material, figure S5. In addition, we report the levels of association between differences in metabolic association and SNPs localized in genes previously reported for BMI [22] (electronic supplementary material, table S4) and SNPs previously reported to influence human serum metabolite levels [15] (electronic supplementary material, table S5).

**Table 2.** Genetic loci associated with differences in metabolite–metabolite correlations between obese and non-obese individuals. Candidate gene, potential candidate gene in the region; Chr, chromosome; Pos, SNP position in NCBI human genome build 36; *p*-value, *p*-value for association between locus and variation in correlation between XL-VLDL/M-VLDL. Significant associations between genetic variants and variation in association between pairs of metabolites were identified using genome-wide significance level threshold (*p* < 5 × 10^−8^/*D*; where *D* = 5).

candidate gene	SNP	Chr	Pos	*p*-value
DOCK	rs1334806	1	62875313	1.32 × 10^−09^
PPP1R12B	rs12743401	1	202476648	1.01 × 10^−13^
OTOF	rs7592040	2	26741551	1.47 × 10^−09^
ABHD5	rs1078248	3	43794357	2.36 × 10^−09^
GRIA1	rs573496	5	152893433	1.94 × 10^−09^
UST	rs2500542	6	149331204	7.95 × 10^−10^
DLC1	rs1454953	8	13315983	6.23 × 10^−10^
DLC1	rs7814428	8	13324439	2.37 × 10^−10^
HEY1	rs2920949	8	80869508	4.14 × 10^−09^
FAM84B	rs7357357	8	127189138	4.49 × 10^−09^
FAM84B	rs10094775	8	127197554	6.50 × 10^−09^
FAM84B	rs4557669	8	127306602	1.97 × 10^−09^
SH2D4B	rs10509433	10	83080528	2.00 × 10^−10^
OR10A6	rs17315588	11	7913911	3.99 × 10^−10^
OR10A6	rs1564632	11	7873288	3.23 × 10^−09^
EED	rs9971532	11	85929490	1.72 × 10^−09^
LDHB	rs1650307	12	21803770	3.11 × 10^−09^
DACH1	rs7981816	13	72642843	5.33 × 10^−09^
NAGPA	rs2302553	16	5056295	3.06 × 10^−09^
NAGPA	rs3815490	16	5060568	9.01 × 10^−10^
ATP4A	rs8106239	19	36085358	9.14 × 10^−10^
BPIFB3	rs2093066	20	31652596	7.81 × 10^−10^
BPIFB3	rs378098	20	31660543	7.46 × 10^−10^
MN1	rs2040699	22	28042532	3.72 × 10^−09^

Several SNPs significantly associated with differences in correlation between total lipids in medium VLDL and total lipids in very-large VLDL are localized in or near a gene previously implicated in the processes related to obesity or associated traits. For instance, we identified SNP rs1334806 which is localized on chromosome 1 near a gene encoding for dedicator of cytokinesis 7 (DOCK7). Variations in this genetic region have been previously found to be implicated in the control of lipid levels [28,29]. Another of the SNPs detected (rs1078248) at chromosome 3p21.33 is localized near a gene encoding for the abhydrolase domain containing five protein (ABHD5/CG1–58). The protein encoded by this gene is known to be linked to triglyceride (TAG) metabolism which is the primary energy source in vertebrates [30]. TAGs are stored in adipose tissue and hydrolysed into fatty acids and glycerol and can be used by body tissues during times of fast or energy deprivation [31]. Mutations in the gene encoding for ABHD5 have been associated with a triglyceride storage disease with impaired long-chain fatty acid oxidation called Chanarin–Dorfman syndrome [32,33]. Despite the fact that ABHD5 is involved in TGA metabolism and highly expressed in adipose tissue, patients with Chanarin–Dorfman syndrome are not obese [34]. No previous association between ABHD5 and obesity-related traits has been reported. Another SNP associated with differences in correlation between total lipids in medium VLDL and very-large VLDL is rs573496 at chromosome 5q33.2. This SNP is localized within a gene encoding for glutamate receptor 1 (GRIA1). Glutamate receptors are protein complexes formed by the combination of four different subunits (GRIA1–GRIA4) and are known to be the main excitatory neurotransmitter receptors in the mammalian brain. Regulation of glutamate receptors is influenced by leptin levels [35].

While none of the SNPs localized within genes previously associated with BMI [22] reach the genome-wide significance threshold (*p* < 5 × 10^−8^/*D*; see the electronic supplementary material, table S4), we observe a relatively high association (*p* < 1 × 10^−4^) between a number of these SNPs and differences in association between total lipids in medium VLDL and very-large VLDL. These SNPs are localized in five obesity-related genetic regions, namely fat mass and obesity-associated protein (rs4784351 with *p* = 3.62 × 10^−5^); transmembrane protein 18 (rs2049480 with *p* = 8.13 × 10^−7^); neurexin-3-alpha (rs8011930 with *p* = 2.67 × 10^−6^; rs8012678 with *p* = 2.66 × 10^−7^; rs8020920 with *p* = 8.53 × 10^−7^ and rs987644 with *p* = 1.07 × 10^−7^); cell adhesion molecule 2 gene (rs9812103 with *p* = 1.70 × 10^−5^); LDL receptor-related protein 1B (rs2049480 with *p* = 8.13 × 10^−7^). In addition, two SNPs localized within genes previously reported to influence human serum metabolite levels [15] show association at significance level *p* < 1 × 10^−4^ with differences in correlation between total lipids in medium VLDL and very-large VLDL (electronic supplementary material, table S5). These two genetic variants correspond to SNP rs560887 (*p* = 8.95 × 10^−5^), which is localized within a gene encoding for glucose-6-phosphatase, catalytic, 2 and SNP rs261336 (*p* = 9.31 × 10^−5^), which is localized within hepatic lipase gene.

## 4. Conclusion

While GWASs have greatly contributed to the identification of genetic variants associated with complex traits, these variants typically appear to explain only a small proportion of the observed variability in phenotypes. In this study, we have proposed the analysis of additional phenotypic variations such as differences in associations between metabolic measures to aid the discovery of genetic factors involved in complex traits. To this end, we have introduced a novel analytical approach for the combined analysis of metabolic and genetic information.

Initially, we examined the relationship between obesity and serum metabolites by performing a differential analysis of metabolic associations. Here, we investigated how metabolic associations differ between non-obese and obese individuals and to what extent these differences in metabolic associations can reveal key features of obesity condition. These findings were therefore interpreted as strong evidence of a relationship between these metabolic associations and obesity. We performed a GWCA where genetic variants were tested for association with metabolic correlations. Unlike standard GWAS, which did not reveal any significant associations, this approach yielded many significant genetic loci. A clear limitation of our study is the lack of a replication cohort for our genetic findings. However, the fact that we found no associations following standard GWAS, but did find a large number of associations using our GWC approach, many of which are implicated in obesity-related processes, is very promising. Moreover, the relatively low *p*-values for some of the previously reported obesity-related gene lend weight to our assertion that the GEMINi method can retrieve useful and biologically meaningful relationships between genetic variants and differences in molecular association. This study therefore highlights the importance of investigating differences in association between metabolites or other phenotypic indicators as an additional phenotype to identify novel loci with key roles in the pathogenesis of complex traits.
